# DOES TAI CHI PROMOTE COGNITIVE FUNCTION WITHOUT THE EFFECTS OF PHYSICAL FUNCTION?: META-REGRESSION APPROACH

**DOI:** 10.1093/geroni/igac059.2892

**Published:** 2022-12-20

**Authors:** Rhayun Song, Moonkyoung Park, Jacqueline Shin, Xianqi Gao, Yuelin Li

**Affiliations:** Chungnam National University, Daejeon, Taejon-jikhalsi, Republic of Korea; Chungnam National University, Daejeon, Taejon-jikhalsi, Republic of Korea; Indiana State University, Terre Haute, Indiana, United States; Chungnam National University, Daejeon, Taejon-jikhalsi, Republic of Korea; Chungnam National University, Daejeon, Taejon-jikhalsi, Republic of Korea

## Abstract

This systematic review aimed to examine the effects of TCQ on cognitive and physical functions in older adults using a meta-regression approach. The systematic search in 13 electronic databases identified 19 randomized studies (n=2365, mean age=70.3 years) published in English (k=17), Korean (k=1) and Chinese (k=1). A review of bias was assessed by two raters according to Cochrane RoB 2.0, resulting in low risk (k=6), some concern (k=12), and one high-risk study. Tai Chi (k=16) and Qigong (k=3) were applied for an average duration of 20.2 weeks. The control groups received either alternative exercise (k=14) or no treatment (k=5). The results of the meta-analysis on 19 RCTs using a random-effects model showed the significant effect of TCQ on cognitive function (Hedges’s g =0.32, 95% CI= 0.18, 0.46) and physical function (Hedges’s g= 0.35, 95% CI= 0.21, 0.49). In addition, meta-regression was used to explore the effect size of TCQ in association with the level of physical function. The effects of TCQ on cognitive function remained significant (Q=38.86, p=.003) when controlling for the effect of physical function in this model (unexplained variance = 0.0199). The coefficient of the physical function was significant (b=0.47, p=.008), showing that 58% of heterogeneity was explained by physical function as a moderator variable. It confirmed that changes in physical function were associated with changes in cognitive function. The findings imply the potential health benefits of TCQ in promoting cognitive function among older adults directly and indirectly through improving physical function.

